# Occurrence of Mineral Oil Hydrocarbons in Omega-3 Fatty Acid Dietary Supplements

**DOI:** 10.3390/foods10102424

**Published:** 2021-10-13

**Authors:** Alessia Arena, Mariosimone Zoccali, Alessandra Trozzi, Peter Q. Tranchida, Luigi Mondello

**Affiliations:** 1 Department of Chemical, Biological, Pharmaceutical and Environmental Sciences, University of Messina, 98168 Messina, Italy; alessia.arena@unime.it (A.A.); alessandra.trozzi@unime.it (A.T.); lmondello@unime.it (L.M.); 2 Department of Mathematical and Computer Science, Physical Sciences and Earth Sciences, University of Messina, 98166 Messina, Italy; 3 Chromaleont s.r.l., c/o Department of Chemical, Biological, Pharmaceutical and Environmental Sciences, University of Messina, 98168 Messina, Italy; 4 BeSep s.r.l., c/o Department of Chemical, Biological, Pharmaceutical and Environmental Sciences, University of Messina, 98168 Messina, Italy; 5 Unit of Food Science and Nutrition, Department of Medicine, University Campus Bio-Medico of Rome, 00128 Rome, Italy

**Keywords:** mineral oil aromatic hydrocarbons, mineral oil hydrocarbons, mineral oil saturated hydrocarbons, multidimensional liquid–gas chromatography, omega-3 fatty acids

## Abstract

Omega-3 fatty acid dietary supplements have become increasingly popular with consumers due to their multiple health benefits. In this study, the presence of mineral oil hydrocarbons (MOH) was investigated in seventeen commercial samples of such supplements, characterized by different formulations. The analyses were performed using on-line liquid chromatography–gas chromatography (with flame ionization detection), which is considered the most efficient method for the determination of MOH in foodstuffs. Analyte transfer was performed by using the retention gap technique, with partially concurrent solvent evaporation. Various degrees of mineral oil saturated hydrocarbon contamination (from 2.4 ppm to 375.7 ppm) were found, with an average value of 49.9 ppm. Different C-number range contaminations were determined, with the >C_25_–≤C_35_ range always found with an average value of 26.9 ppm. All samples resulted free of mineral oil aromatic hydrocarbons, except for two samples in which a contamination was found at the 9.9 and 6.6 ppm levels, respectively.

## 1. Introduction

Mineral oil hydrocarbons (MOH) have been defined by the European Food Safety Authority (EFSA) as molecules containing from 10 to about 50 carbon atoms, and are divided into two classes: mineral oil saturated (MOSH) and aromatic hydrocarbons (MOAH) [1]. MOH can be found in food as a result of various events, such as environmental contamination, intentional use during food production, and transfer via food packaging.

The first paper focused on the analysis of MOH contamination in food was published in 1991 [2]. Since then, several studies have been published involving many types of food. For instance, Purcaro et al. in 2013 investigated the presence of MOSH in different food samples (rice, icing sugar, pasta, and olive oil), detecting amounts ranging between < limit of quantification (LoQ) and 65.2 ppm [3]. In 2016, Moret et al. determined the mineral oil content in cereal-based products of different compositions [4]; among the different products analyzed, egg pasta had on average the highest total MOSH level (15.9 ppm), followed by cakes (10.4 ppm), and bread (7.5 ppm). The highest concentration of MOAH was found in an egg pasta (3.6 ppm) and in a milk bread (3.6 ppm) sample. In the same year, eleven vegetable oils (four different types) were analyzed by Zoccali et al., with detected amounts of MOSH ranging between 4 (extra virgin olive oil) and 2540 ppm (sunflower oil) and amounts of MOAH ranging between <LoQ and 356 ppm (sunflower oil) [5]. In 2018, Canavar et al. carried out research focused on the analysis of MOH in dry foodstuffs packed in recycled paperboard [6]. The MOSH concentration ranged from 0.1 to 42.9 ppm, while the MOAH content ranged from “not detected” to 2.7 ppm. A further study, focused on the analysis of packed food samples from supermarkets in Belgium, was performed by Van Heyst et al. in 2018. A MOSH contamination was detected in 142 samples up to 84.8 ppm, and 23 samples were contaminated by MOAH with a concentration of up to 2.2 ppm [7]. In 2020, Liu et al. analyzed the MOH in ten milk powder samples, finding a content of MOSH and polyolefin oligomeric saturated hydrocarbons in the 0.61–5.46 ppm range [8]. In 2020, Zoccali et al. and Stauff et al. analyzed several kinds of vegetable oils and fats, detecting high levels of MOSH and MOAH, especially in cocoa butter and corn oil samples [9,10].

In recent years, the consumption of omega-3 fatty acid (FA) dietary supplements has increased considerably, due to their multiple health benefits. In fact, as reported by the *Omega-3 Supplements Market Size, Share & Trends Analysis Report* [11], in 2019 the global market was valued at USD 5.18 billion, and is expected to grow at a compound annual growth rate of 8.4%, from 2020 to 2027. Long-chain omega-3 polyunsaturated fatty acids (PUFA), eicosapentaenoic acid (EPA) and docosahexaenoic acid (DHA) are biologically active lipids, which play essential roles in many physiological processes, most notably in neuronal functioning and inflammatory pathways [12,13,14]. Through antioxidant properties, omega-3 PUFA may improve vascular functions and reduce atherosclerotic damage [15]. Moreover, EPA and DHA may exert anti-cancer activity, either alone or with conventional therapies [16].

Nowadays, omega-3 FA dietary supplements can contain fish oil (as a generic term), krill oil, cod liver oil, and also vegetarian products that contain algal oil.

In 2010, the Food and Agriculture Organization of the United Nations (FAO) released recommendations for adult males and non-pregnant/non-lactating females to consume 250 mg of EPA + DHA per day [17]. According to the U.S. Department of Health and Human Services, a typical fish oil supplement provides about 1 g of fish oil, containing 180 mg EPA and 120 mg DHA, even though doses vary widely [18].

Although seafood can contain various levels of toxic heavy metals [19], omega-3 FA dietary supplements have not been found to contain such contaminants, while the presence of polychlorinated biphenyls (PCB) has been determined to be below the legal limit [20], probably because they were removed via distillation/deodorization steps [21]. However, in 2019, Matsuo et al. found that 17 of the 26 fish oil products analyzed were contaminated with PCB, with a median concentration of 2.2 ppb [22].

The aim of the present study was to investigate the MOH contamination in omega-3 FA dietary supplements, since, to the best of the authors’ knowledge, no previous research has been published on such a topic. Following the EU guidance [23], the analyses were performed on-line by using liquid chromatography hyphenated with gas chromatography (LC–GC), equipped with a flame ionization detector (FID) and a lab-made Y-interface [24].

## 2. Materials and Methods

### 2.1. Samples and Sample Preparation

Seventeen omega-3 FA dietary supplement samples were prepared as follows: 250 mg of oil sample was weighed, 25 µL of the internal standard (IS) mixture was added, and both were diluted to a final volume of 1 mL with *n*-hexane. For samples contained in soft gel capsules, the capsules were broken, and the oil portion was used while the capsule portion was discarded. All samples were analyzed in triplicate (*n* = 3).

All analyzed samples are reported in Table 1, along with their compositions, the formulation type, the MOSH and MOAH contamination levels, and the CV% values.

### 2.2. Chemicals

*n*-Hexane (for high pressure liquid chromatography (HPLC), gas chromatography (GC), grade ≥ 97%) and dichloromethane (CH_2_Cl_2_) (grade ≥ 99.9%) were acquired from Merck Life Science (Merck KGaA, Darmstadt, Germany). For the MOSH and MOAH separation and quantification, 10 standard compounds were used (stock solution): *n*-undecane (*n*-C_11_), *n*-tridecane (*n*-C_13_), bicyclohexyl (CyCy), 5-α-cholestane (Cho), 1-methylnaphthalene (1MN), 2-methylnaphthalene (2MN), *n*-pentylbenzene (5B), perylene (Per), 1,3,5-tri-tert-butylbenzene (TBB), 1,4-bis (2-ethylhexyl) benzene (DEHB). C_11_, CyCy, 1MN, 2MN, 5B, TBB, and DEHB were at a concentration of 300 μg mL^−1^ each. C_13_ was at a concentration of 150 μg mL^−1^, while Cho and Per were at a concentration of 600 μg mL^−1^. An IS mixture was prepared by combining 0.1 mL of the stock solution with *n*-hexane to a final volume of 3 mL.

For discrimination evaluation, the following mixture, containing 10 standard compounds, was used: *n*-decane (*n*-C_10_), *n*-C_11_, *n*-C_13_, *n*-hexadecane (*n*-C_16_), *n*-eicosane (*n*-C_20_), *n*-tetracosane (*n*-C_24_), *n*-pentacosane (*n*-C_25_), *n*-pentatriacontane (*n*-C_35_), *n*-tetracontane (*n*-C_40_), *n*-pentacontane (*n*-C_50_), all at a concentration of 100 μg mL^−1^ (Merck Life Science).

### 2.3. LC-GC Analyses

The analyses were carried out using an LC–GC system equipped with a lab-constructed Y-interface [24].

The instrument was composed of a Shimadzu Prominence LC-20A system (Kyoto, Japan) equipped with: a CBM-20A communication bus module, a DGU-20A on-line degasser, two LC-20AD dual-plunger parallel-flow pumps, a SIL-20AC autosampler, a CTO-20A column oven and an SPD-M30A photodiode array detector. The exit of the latter was connected, by a capillary tube, to port 2 of an FCV-20AH2 six-port two-position switching valve, connected and controlled by the LC system. Port 3 of the valve was directed to the waste, while port 1—through a fused silica capillary—was connected to a GC2010 gas chromatograph (Shimadzu) equipped with a split/splitless injector and an FID.

The LC–GC transfer process was performed using the retention gap technique, with partially concurrent solvent evaporation; two “Y” connectors were located inside the GC oven. The first was connected to port 1 of the switching valve, by means of a fused silica capillary, to the injector, for carrier gas feeding, and to a deactivated uncoated pre-column. The second was connected to the previously mentioned deactivated uncoated pre-column, to a three-port two-position valve (Shimadzu), employed for solvent evaporation, and to the analytical column. For detailed information, the reader is directed to a previously published paper [24].

The LC conditions were as follows: A LiChrospher Si 60 Å column of the dimensions 25 cm × 2.1 mm I.D. × 5 µm *d_p_* (Merck Life Science) was employed at a flow of 0.3 mL min^−1^ in the normal-phase mode. The following gradient elution was employed: 100% hexane from start to 0.8 min, increasing up to 30% of CH_2_Cl_2_ in 0.7 min, for 8 min. The sample injection volume was 100 µL.

The MOSH fraction was transferred from 2.00 to 3.50 min, and the MOAH fraction was transferred from 4.5 to 8.1 min. After the 10 min run, the column was washed in backflush with CH_2_Cl_2_ and then reconditioned with *n*-hexane. GC conditions were as follows: a 10 m × 0.53 mm I.D. deactivated uncoated pre-column was connected to the analytical column, which was an SLB-5ms (silphenylene polymer, virtually equivalent in polarity to poly (5% diphenyl/95% methylsiloxane)) of dimensions 20 m × 0.25 mm I.D. × 0.10 µm *d_f_* (Merck Life Science).

The GC oven temperature program was: 60 °C (6 min for MOSH and 8 min for MOAH) to 360 °C at 10 °C min^−1^. Carrier gas, helium, was supplied at an initial pressure of 80.2 kPa (2.5 min) (for solvent removal), increased up to 500 kPa at 200 kPa min^−1^; for MOAH analysis the initial pressure was held at 80.2 kPa for 5.0 min. The initial average linear velocity was 130 cm s^−1^. The FID (360 °C) sampling frequency was 8.3 Hz.

### 2.4. Method Validation

The IS mixture was used to both verify the correct transfer of the two fractions and the quantification of MOSH and MOAH humps.

Compound CyCy was used as internal standard for MOSH quantification because it is not found in relevant quantities in mineral oils and packaging.

Compound C_11_, added in the same amount as CyCy, was used to verify the loss of CyCy. Compound C_13_, used as second verification standard, eluted immediately after CyCy and created a typical pair of peaks that is easily recognized. Finally, Cho was used to mark the end of the MOSH fraction. DEHB and Per were used as markers of the beginning and the end of the MOAH fraction, respectively. Although TBB has been historically employed to control the beginning of the MOAH fraction, DEHB is a more suitable marker, because it allows a double check in both the LC and GC runs. Compound 5B was used to monitor the loss of volatiles, while 1MN was used for the quantification of the MOAH hump. 1MN and 2MN, in the same amount, form a pair of easily recognized peaks.

Quantification was carried out using an FID, which provides the same response factor for all the hydrocarbons of interest, allowing simplification of the quantification procedure by the use of appropriate internal standards, specifically CyCy for the MOSH and 1MN for the MOAH [25]. The area of each hump was integrated from valley to valley by using the “manual integration” function of the GC-FID software, while internal standards and endogenous hydrocarbons, present on the top of the hump, were subtracted from the results. To optimize quantification, a “blank” analysis was acquired every day.

Sub-fractions of MOSH and MOAH were determined by injection of a mixture of 10 reference alkanes (from C_10_ to C_50_). Intermediate precision was evaluated across a period of 48 days obtaining a CV (coefficient of variation) value of 10% (within the value of 20% reported by the EU guidelines for fat/oils samples) [23]; in total, 6 samples were considered (*n* = 3), prepared on different days.

In order to obtain an LoQ of 2 mg kg^−1^, as required by EU guidance for oil and fat foods [23], an injection volume of 100 µL was used (considering the dilution factor, an absolute amount of 25 mg of oil was injected).

## 3. Results and Discussion

Mineral oil contamination in food is becoming of great concern due to its potential adverse health effects. As reported by EFSA [1], MOSH with carbon numbers between 16 and 35 may accumulate in different parts of the human body such as adipose tissue, lymph nodes, spleen and liver, while MOAH have mutagenic capabilities due to their 3–7 aromatic rings.

In 2014, Barp et al. analyzed MOH in the subcutaneous abdominal fat tissue, mesenteric lymph nodes (MLN), spleen, liver, and lungs of 37 human subjects [26]. The highest concentrations of MOSH were found in MLN and spleen, with 1390 and 1400 mg/kg, respectively. No MOAH were detected.

In the past few decades, omega-3 FA dietary supplements have become increasingly popular and have spread widely in the field of natural health products. For such a reason, in the present study seventeen commercial products, prepared using fish, vegetable and microalgae oils, were analyzed. Of these, 14 samples were contained in soft gel capsules, while three samples were liquid.

Quantitative data relative to the MOSH and the MOAH analyses are reported in Table 1.

**Figure 1:**
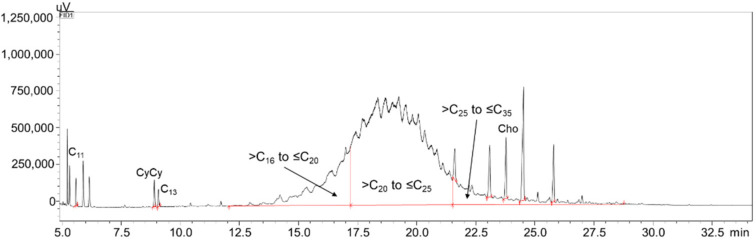
LC-GC expansion of the MOSH fraction of sample 2, along with IS and C-fractions.

Figure 1 reports the most contaminated sample (sample 2), with a MOSH level of 375.7 ppm. As required by the EU guidance [23], the contamination values must be expressed in sub-fractions, defined by the position of the elution signals of reference alkanes from the GC column. In this case, the hump was in the >C_16_–≤C_35_ range (60.9 ppm in the >C_16_–≤C_20_ range, 278.8 ppm in the >C_20_–≤C_25_ range, and 36.0 ppm in the >C_25_–≤C_35_ range).

**Figure 2:**
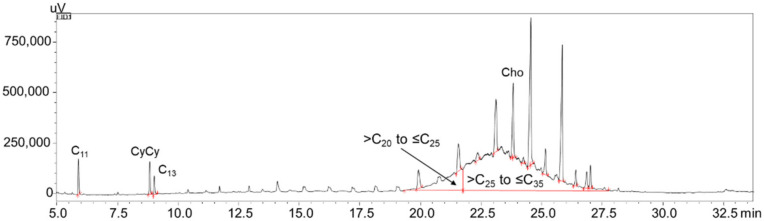
LC–GC expansion of the MOSH fraction of sample 5, along with IS and C-fractions.

The MOSH fraction of sample 5 is shown in Figure 2. In this sample, the contamination was moderate, at the 92.9 ppm level (13.3 ppm in the >C_20_–≤C_25_ range, 79.6 ppm in the >C_25_–≤C_35_ range); sample 12 was also characterized by a moderate contamination at the 65.4 ppm level (6.3 ppm in the >C_20_–≤C_25_ range, 55.6 ppm in the >C_25_–≤C_35_ range, 3.5 ppm in the >C_35_–≤C_40_ range).

**Figure 3:**
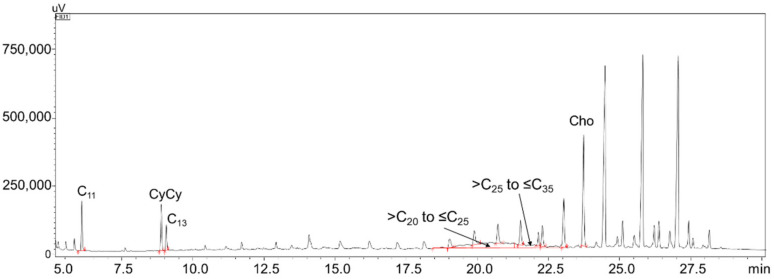
LC–GC expansion of the MOSH fraction of sample 3, along with IS and C-fractions.

Sample 13 presented a contamination at the 73.5 ppm level, with the MOSH hump fully centered in the >C_25_–≤C_35_ range. A very low amount of MOSH was detected in samples 3, 8, 11, 14, and 16, namely 6.3, 2.4, 4.2, 3.1, and 3.8 ppm, respectively. One of the lowest contaminated samples (sample 3) is shown in Figure 3. Considering all samples analyzed, the CV was between <1 and 8%.

The MOSH contamination for each C-fraction, for the analyzed samples, is shown in Table 2. As can be seen, all the samples subjected to analysis presented a contamination in the >C_25_–≤C_35_ range, with an average value of 26.9 ppm; six samples were characterized by a contamination in the >C_20_–≤C_25_ range, with an average value of 50.8 ppm; a contamination in the >C_35_–≤C_40_ range was found in nine samples, with an average value of 2.6 ppm. Only sample 2 presented a contamination in the >C_16_–≤C_20_ range (60.9 ppm). Finally, contamination in the >C_40_–≤C_50_ range was present only in samples 1 and 6, at low concentration levels, specifically 1.8 and 0.6 ppm, respectively.

Considering the ten samples prepared exclusively with fish oil, the contamination in the >C_25_–≤C_35_ range had an average value of 30.6 ppm, with a minimum of 2.4 ppm and a maximum of 79.6 ppm; the >C_35_–≤C_40_ range was characterized by an average value of 1.7 ppm, with a minimum of 0.4 ppm and a maximum of 4.7 ppm.

Taking into consideration the samples made up of both vegetable and fish oils, the >C_20_–≤C_25_ range had an average value of 96.8 ppm, with a minimum of 5.3 ppm and a maximum of 278.8 ppm; the >C_25_–≤C_35_ range was characterized by an average value of 23.3 ppm, with a minimum of 1 ppm and a maximum of 55.6 ppm; the >C_35_–≤C_40_ range presented an average value of 4.7 ppm, with a minimum of 0.2 ppm and a maximum of 10.3 ppm.

Finally, MOAH contamination was only found in samples 7 and 13 (both fish), at the 9.9 and 6.6 ppm levels, respectively.

Oral ingestion through dietary intake is considered the major source of MOH exposure for consumers. Considering the average value of MOSH contamination found in the 17 analyzed samples (49.9 ppm), a daily amount of 1 g of oil would provide an average ingestion amount of MOSH of 49.9 µg. In the *Scientific Opinion on Mineral Oil Hydrocarbons in Food*, EFSA reported that MOSH absorption (through the portal and/or the lymphatic system) decreases by increasing carbon numbers, varying from 90% for C_14_–C_18_ to 25% for C_26_–C_29_, decreasing further until above C_35_ when it becomes negligible [1]. Furthermore, as reported by Barp et al., the maximum MOSH concentrations varied between C_25_ and C_28_ in human livers and spleens [27]. In the present research, all the analyzed samples presented a MOSH contamination in the >C_25_–≤C_35_ range, and are thus the biggest concern for human health based on liver and spleen accumulation. Moreover, two samples were also contaminated by MOAH (albeit at low concentrations), which as previously mentioned present potential mutagenic and carcinogenic effects.

## 4. Conclusions

All the commercial samples subjected to analysis were characterized by MOSH contamination, at various levels from 2.4 to 375.7 ppm; only two samples also presented a MOAH contamination.

The bioaccumulation of MOSH with related inflammatory activity and the carcinogenic potential of MOAH represent the main toxicological problems associated with MOH.

In relation to the obtained results in this study, it would be advisable that the pharmaceutical industries pay more attention to the possible presence of these contaminants, in addition to the determination of other xenobiotics and oxidation-related products (including heavy metals, peroxide, and anisidine values) in the raw materials. Furthermore, attention should be focused on extraction techniques and possible migration from the gel capsules and from the packaging. Regarding the possible migration from the gel capsules, a future study will be focused on different materials employed for the formulations.

## Figures and Tables

**Table 1:** List of the analyzed samples along with composition, formulation, MOSH and MOAH contamination levels, and CV% values.

Sample N°	Composition (Oil)	Formulation	MOSH (ppm)	CV% (MOSH)	MOAH (ppm)
1	Vegetable + fish	Capsule	33.0	5	<LoQ
2	Vegetable + fish	Capsule	375.7	3	<LoQ
3	Vegetable + fish	Capsule	6.3	2	<LoQ
4	Fish	Capsule	43.9	3	<LoQ
5	Fish	Capsule	92.9	4	<LoQ
6	Fish	Liquid	14.4	8	<LoQ
7	Fish	Capsule	42.3	2	9.9
8	Fish	Capsule	2.4	8	<LoQ
9	Fish	Capsule	18.4	1	<LoQ
10	Fish	Capsule	16.3	1	<LoQ
11	Fish	Capsule	4.2	3	<LoQ
12	Fish + vegetable	Capsule	65.4	4	<LoQ
13	Fish	Capsule	73.5	<1	6.6
14	Fish + vegetable	Liquid	3.1	6	<LoQ
15	Fish	Capsule	20.2	<1	<LoQ
16	Microalgae + vegetable	Liquid	3.8	2	<LoQ
17	Fatty acid methyl esters	Capsule	32.8	1	<LoQ

**Table 2:** Analyzed samples along with MOSH and MOAH C-fractions, and concentrations.

	MOSH			MOAH
Sample N°	C-Fraction	ppm	C-Fraction	ppm
1	C_25_–C_35_ C_35_–C_40_ C_40_–C_50_	20.9 10.3 1.8	-	-
2	C_16_–C_20_ C_20_–C_25_ C_25_–C_35_	60.9 278.8 36.0	-	-
3	C_20_–C_25_ C_25_–C_35_	5.3 1.0	-	-
4	C_25_–C_35_ C_35_–C_40_	42.5 1.4	-	-
5	C_20_–C_25_ C_25_–C_35_	13.3 79.6	-	-
6	C_25_–C_35_ C_35_–C_40_ C_40_–C_50_	9.1 4.7 0.6	-	-
7	C_25_–C_35_ C_35_–C_40_	41.4 0.8	C_25_–C_35_	9.9
8	C_25_–C_35_	2.4	-	-
9	C_25_–C_35_	18.4	-	-
10	C_25_–C_35_	16.3	-	-
11	C_25_–C_35_ C_35_–C_40_	3.8 0.4	-	-
12	C_20_–C_25_ C_25_–C_35_ C_35_–C_40_	6.3 55.6 3.5	-	-
13	C_25_–C_35_	73.5	C_25_–C_35_	6.6
14	C_25_–C_35_ C_35_–C_40_	2.9 0.2	-	-
15	C_25_–C_35_ C_35_–C_40_	19.1 1.1	-	-
16	C_20_–C_25_ C_25_–C_35_	0.3 3.5	-	-
17	C_20_–C_25_ C_25_–C_35_ C_35_–C_40_	0.9 31.3 0.6	-	-
